# Integrative taxonomic analyses reveal first country records of *Occidozygashiwandashanensis* Chen, Peng, Liu, Huang, Liao & Mo, 2022 and *Hylaranalatouchii* (Boulenger, 1899) (Anura, Dicroglossidae, Ranidae) from Vietnam

**DOI:** 10.3897/BDJ.11.e109726

**Published:** 2023-10-13

**Authors:** Tung Thanh Tran, Chung Van Hoang, Anh Mai Luong, Truong Quang Nguyen, Thomas Ziegler, Cuong The Pham

**Affiliations:** 1 Vinh Phuc College, Vinh Phuc Province, Vietnam Vinh Phuc College Vinh Phuc Province Vietnam; 2 Forest resources and environment center, Hanoi, Vietnam Forest resources and environment center Hanoi Vietnam; 3 Institute of Ecology and Biological Resources, Vietnam Academy of Science and Technology, Hanoi, Vietnam Institute of Ecology and Biological Resources, Vietnam Academy of Science and Technology Hanoi Vietnam; 4 Graduate University of Science and Technology, Vietnam Academy of Science and Technology, Hanoi, Vietnam Graduate University of Science and Technology, Vietnam Academy of Science and Technology Hanoi Vietnam; 5 Cologne Zoo, Cologne, Germany Cologne Zoo Cologne Germany; 6 Institute of Zoology, University of Cologne, Cologne, Germany Institute of Zoology, University of Cologne Cologne Germany

**Keywords:** *Occidozygashiwandashanensis*, *Hylaranalatouchii*, genetic divergence, morphology, new records

## Abstract

**Background:**

*Occidozygashiwandashaensis* was recently discovered from Guangxi Province of China. *Hylaranalatouchii* is a widespread species in southern China, including Hong Kong and Taiwan. Both species are expected to be found in the border areas between Vietnam and China; however, no records of these frogs have been documented from Vietnam so far.

**New information:**

We record two species of amphibians for the first time from Vietnam, namely *Occidozygashiwandashaensis* from Bac Giang Province and *Hylaranalatouchii* from Hai Phong City and Quang Ninh Province in northern Vietnam. Morphologically, the Vietnamese representatives of *O.shiwandashanensis* resemble the type series from China. The specimens of *H.latouchii* from Vietnam slightly differ from the type series from China by having a larger size (SVL 48.6–51.7 mm in males, SVL 58.4 mm in the females vs. 36.0–40.0 mm in males, 42.0–53.0 mm in females). Genetic distances between the Vietnamese records and the type specimens of *O.shiwandashanensis* from China varied from 0 to 1.5% (16S gene). Genetic divergences between the Vietnamese records and *H.latouchii* from the type locality were 2.0–2.6% (16S gene). In addition, morphological data and natural history notes of the aforementioned species are provided, based on the new records from Vietnam. ­­

## Introduction

The border region between Vietnam and China is characterised by a geologically and environmentally complex, a mixture of granite and limestone, uplands and delta, jagged peaks and humid lowlands and tropical and subtropical species (Sterling et al. 2006). The forests in the border region between China and Vietnam harbour a high level of herpetofaunal diversity with a remarkable number of new discoveries in particular from northern Vietnam in the last decade, viz. *Gracixaluswaza* Nguyen, Le, Pham, Nguyen, Bonkowski and Ziegler, 2013; *Odorranamutschmanni* Pham, Nguyen, Le, Bonkowski and Ziegler, 2016; *Limnonectesquangninhensis* Pham, Le, Nguyen, Ziegler, Wu and Nguyen, 2017; *Gracixalussapaensis* Matsui, Ohler, Eto and Nguyen, 2017; *Boulenophryshoanglienensis* (Tapley, Cutajar, Mahony, Nguyen, Dau, Luong, Le, Nguyen, Nguyen, Portway, Luong and Rowley, 2018); *Zhangixalusfranki* Ninh, Nguyen, Orlov, Nguyen and Ziegler, 2020; *Leptobrachellagraminicola* Nguyen, Tapley, Nguyen, Luong and Rowley, 2021; *Thelodermakhoii* Ninh, Nguyen, Nguyen, Hoang, Siliyavong, Nguyen, Le, Le and Ziegler, 2022; *Microhylahmongorum* Hoang, Nguyen, Phan, Pham, Ninh, Wang, Jiang, Ziegler and Nguyen, 2022; *Gracixalustruongi* Tran, Pham, Le, Nguyen, Ziegler and Pham, 2023 (Frost 2023, Nguyen et al. 2013, Pham et al. 2016b, Pham et al. 2017, Matsui et al. 2017, Tapley et al. 2018, Ninh et al. 2020, Ninh et al. 2022, Nguyen et al. 2021, Hoang et al. 2022, Tran et al. 2023). In the last ten years, several new species of amphibians have been described in the border region, viz. *Microhylahmongorum* Hoang, Nguyen, Phan, Pham, Ninh, Wang, Jiang, Ziegler and Nguyen, 2022 (Hoang et al. 2022, Wu et al. 2023); *Limnonectesnguyenorum* McLeod, Kurlbaum and Hoang, 2015 (McLeod et al. 2015, Liu et al. 2022); *Amolopswenshanensis* Yuan, Jin, Li, Stuart and Wu, 2018 (Yuan et al. 2018, Pham et al. 2020b); *Odorranafengkaiensis* Wang, Lau, Yang, Chen, Liu, Pang and Liu, 2015 (Wang et al. 2015, Pham et al. 2020a); *Odorranalipuensis* Mo, Chen, Wu, Zhang and Zhou, 2015 (Mo et al. 2015, Pham et al. 2016a); and *Tylototritonziegleri* Nishikawa, Matsui and Nguyen, 2013 (Nishikawa et al. 2013, Ye et al. 2017).

As a result of our recent fieldwork in north-eastern Vietnam, we recorded two amphibian species for the first time for Vietnam, viz. *Occidozygashiwandashanensis* Chen, Peng, Liu, Huang, Liao and Mo, 2022, a recently-described species from Shiwandashan Mountain, Fangcheng, Guangxi, China (Chen et al. 2022) and *Hylaranalatouchii* (Boulenger, 1899), a species previously known only from southern China (Zhejiang, Fujian, Guangxi, Hong Kong, Guangdong, Hunan, Jiangxi, Jiangxu and Anhui), including Taiwan (Frost 2023).

## Materials and methods

### Sampling

**Figure 1.**
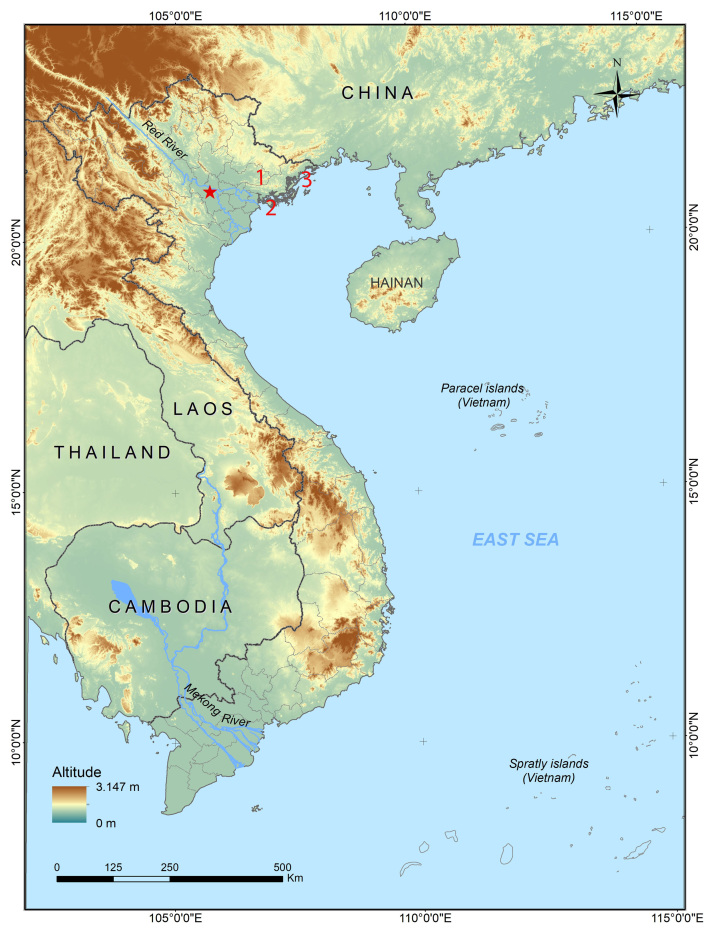
Map showing the survey sites in northern Vietnam: (1) Bac Giang Province, (2) Hai Phong City and (3) Quang Ninh Province. Red star: Hanoi Capital.

Field surveys were conducted in Tay Yen Tu Nature Reserve, Bac Giang Province in June 2007 and in May 2015; in Bai Tu Long National Park, Quang Ninh Province in May 2011, in June 2017 and in June 2023; and in Cat Ba National Park, Hai Phong City in July 2020 (Fig. 1). The coordinates (WGS 84) and elevations were determined by using the GPS Garmin 60CX. Amphibians were collected between 19:00 h and 23:00 h. After taking photographs of the individuals in life, frogs were anaesthetised and euthanised in a closed vessel with a piece of cotton wool containing ethyl acetate (Simmons 2002), fixed in 80% ethanol for four hours, then later transferred to 70% ethanol for permanent storage. For molecular analysis, tissue samples of muscle and liver were preserved separately in 95% ethanol. Preserved specimens were deposited in the collection of the Institute of Ecology and Biological Resources (IEBR), Hanoi, Vietnam.

### Molecular analysis

One sample of *Occidozyga* and two samples of *Hylarana* were amplified fo r ~ 560 base pairs length fragment of the 16S rRNA mitochondrial gene (Suppl. material 1). Tissue samples were extracted using PureLink™ RNA Micro Scale Kit (Thermo Fisher Scientific company), following the manufacturers’ instructions. Total DNA was amplified using PCR Applied Biosystems; the PCR volume consisted of 25 μl, including 12 μl of Mastermix, 6 μl of water, 1 μl of each primer at concentration of 10 pmol/μl and 5 μl of DNA. Primers used in PCR and sequencing were as follows: LR–N–13398 (5’–CGCCTGTTTACCAAAAACAT –3’; forward) and LR–J 12887 (5’–CCGGTCTGAACTCAGATCACGT –3’; reverse) (Simon et al. 1994). PCR conditions: 94°C for 5 minutes of initial denaturation; with 35 cycles of denaturation at 94°C for 30 s, annealing at 56°C for 30 s and extension at 72°C for 45 s; and the final extension at 72°C for 7 minutes. PCR products were sent to Apical Scientific company for sequencing (https://apicalscientific.com). The obtained sequences were deposited in GenBank under the accession numbers R656682 of *Occidozyga* sample and OR656680-OR656681 of *Hylarana* samples.

In addition to the sequence of the newly-collected sample of *Occidozyga* from Vietnam, we used 14 available sequences of 16S rRNA of 11 species of *Occidozyga* from GenBank (Chen et al. 2022) for phylogenetic analyses. Sequences of *Limnonectesjarujini* and *Ingeranatenasserimensis* were included in the analysis as outgroups. Locality information and accession numbers for all sequences included in the analysis can be found in Suppl. material 1.

In addition to the two sequences of the newly-collected samples of *Hylarana* from Vietnam, we used 29 available sequences of 16S rRNA of seven species of *Hylarana* from GenBank for phylogenetic analyses. Sequences of *Babinaholsti* were included in the analysis as an outgroup. Locality information and accession numbers for all sequences included in the analysis can be found in Suppl. material 1.

Chromas Pro software (Technelysium Pty Ltd., Tewantin, Australia) was used to edit the sequences, which were aligned using the ClustalW (Thompson et al. 1997) option in MEGA 7.0 (Kumar et al. 2016) with default parameters and subsequently optimised manually in BioEdit 7.0.5.2 (Hall 1999). Locality information and GenBank accession numbers for all new sequences in this study can be found in Suppl. material 1. Pairwise comparisons of uncorrected sequence divergences (p-distance) were calculated with MEGA 7.0 (Kumar et al. 2016) where the outgroup was excluded. Variance was estimated using the bootstrap method with 1000 replicates using nucleotide substitution, while gap/missing data were treated via pairwise deletion.

Phylogenetic trees were constructed using Maximum Likelihood (ML) and Bayesian Inference (BI). Prior to ML and Bayesian phylogenetic analyses, we chose the optimum substitution models for entire sequences using Kakusan 4 (Tanabe 2011), based on the Akaike Information Criterion (AIC). The BI was performed in MrBayes 3.2 (Ronquist et al. 2012). The BI summarised two independent runs of four Markov Chains for 10,000,000 generations. A tree was sampled every 100 generations and a consensus topology was calculated for 70,000 trees after discarding the first 30,001 trees (burn-in = 3,000,000). We checked parameter estimations and convergence using Tracer version 1.5 (Rambaut and Drummond 2009). The strength of nodal support in the ML tree was analysed using non-parametric bootstrapping with 1000 replicates. We regarded tree nodes in the ML tree with bootstrap values of 75% or greater as sufficiently resolved (Hillis and Bull 1993, Huelsenbeck and Hillis 1993) and nodes with a BPP of 95% or greater as significant in the BI analysis (Leaché and Reeder 2002).

### Morphological examination

Measurements were taken on preserved specimens with a digital caliper to the nearest 0.1 mm. The following abbreviations were used: SVL = snout-vent length, HL = head length (measured as a parallel line with the vertebral column from posterior margin of mandible to tip of snout), HW = maximum head width (across angles of jaws), RL = rostral length (from anterior corner of orbit to tip of snout), NS = distance from nostril to the tip of snout, EN = distance from anterior corner of orbit to the nostril, IND = internarial distance, IOD = interorbital distance, ED = eye diameter, UEW = maximum width of upper eyelid, MN = posterior margin of mandible to nostril, MFE = posterior margin of mandible to anterior corner of orbit, MBE = posterior margin of mandible to posterior corner of orbit; DAE = distance between anterior corners of orbits, DPE = distance between posterior corners of orbits, TD = tympanum diameter, TYE = distance from anterior margin of tympanum to posterior corner of orbit, FLL = forearm length, from elbow to base of outer palmar tubercle, HAL = hand length, from base of outer palmar tubercle to tip of third finger, FL1–4 = Finger length I–IV, NPL = nuptial pad length, FeL = femur length (from vent to knee), TbL= tibia length (from knee to tarsus), TbW = maximum tibia width, FoL = foot length (from tarsus to the tip of fourth toe), TL1–5 = toe length I–V. For webbing formula, we followed Glaw and Vences (2007). Sex was determined by the presence of nuptial pads and based on gonadal inspection.

## Data resources

**Figure 2.**
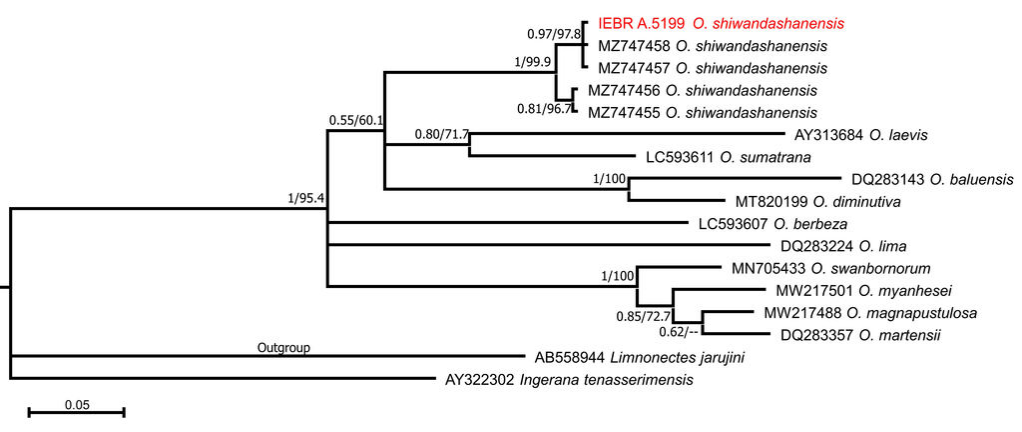
The Bayesian Inference (BI) tree of *Occidozyga*, based on the partial 16S rRNA mitochondrial gene. Values at nodes correspond to BI/ML support values, respectively.

The aligned 16S dataset contained a total of 560 nucleotide base pairs (bp) in length, with 269 variable positions and 176 parsimony informative sites (including outgroups). The BI and ML analyses showed consistent topology (Fig. 2). The results indicated that the monophyly of *Occidozyga* was strongly supported and in agreement with results of Chen et al. (2022). The specimen collected from Bac Giang Province of Vietnam, clustered with the specimens (including type specimens) of *O.shiwandashanensis* from China (Fig. 2). Genetic divergence between the specimen from Vietnam and the type specimens of *O.shiwandashanensis* is approximately 1.5% (Suppl. material 2). It is comparable to the interspecific genetic divergence (uncorrected *p*-distance) between the type samples of *O.shiwandashanensis* which is up to 1.5% (Suppl. material 2). Morphologically, the specimen from Bac Giang Province shows a similar appearance compared with the original description of *O.shiwandashanensis*. Therefore, we considered the population from Bac Giang, Vietnam to be conspecific with *O.shiwandashanensis*.

**Figure 3.**
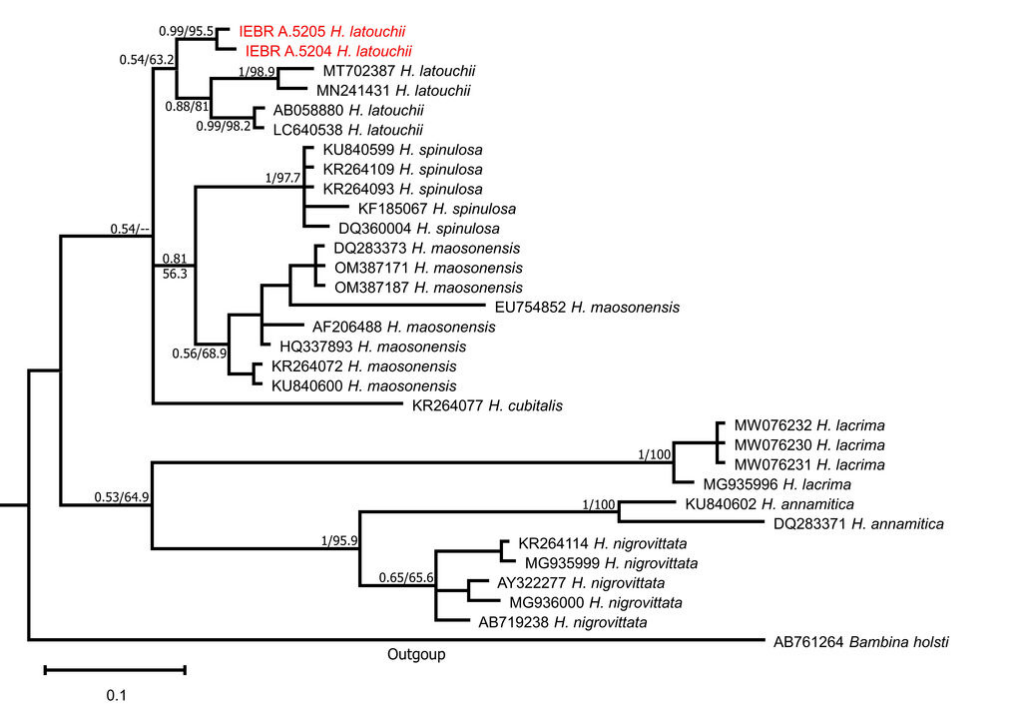
The Bayesian Inference (BI) tree of *Hylarana*, based on the partial 16S rRNA mitochondrial gene. Values at nodes correspond to BI/ML support values, respectively.

The aligned 16S dataset contained a total of 564 nucleotide base pairs (bp) in length, with 100 variable positions and 84 parsimony informative sites (including outgroups). The BI and ML analyses showed consistent topology (Fig. 3). The results indicated that the monophyly of *Hylarana* was strongly supported and two samples from Hai Phong City and Quang Ninh Province were closest to a sample which was collected from Jiulongshan National Nature Reserve, Zhejiang, China by Sun et al. (2021) (voucher specimen LSU20200422001ZL, GenBank accession number MT702387), a sample that was collected from Jinggangshan, Jiangxi Province in China by Xiao et al. (2019) (GenBank accession number MN241431) and a sample that was collected from Taiwan by Sumida et al. (2003) (GenBank accession number AB058880). The specimens collected from Hai Phong City and Quang Ninh Province in Vietnam clustered with those of *H.latouchii* from China (Fig. 3). Genetic divergences between the specimens from Vietnam and the type series of *H.latouchii* were 2.0–2.6% (Suppl. material 3). It is comparable to interspecific genetic divergence (uncorrected *p*-distance) between the samples of *H.latouchii* from China which varied from 0–2% and the samples of *H.nigrovittata* which varied from 0.2–2.8% (Suppl. material 3). Morphologically, the specimens from Quang Ninh Province show a similar appearance compared to the original description of *H.latouchii*. Therefore, we considered the frog population from Hai Phong and Quang Ninh, Vietnam to be conspecific with *H.latouchii*.

## Taxon treatments

### Occidozyga shiwandashanensis

Chen, Peng, Liu, Huang, Liao and Mo, 2022

AECFCCC3-F598-574B-9A72-F6ED7EC0E670

#### Materials

- **Type status:** Other material. **Occurrence:** catalogNumber: IEBR A.5199; individualCount: 1; sex: female; lifeStage: adult; occurrenceID: 82BB7128-5289-5DB2-8D23-EA77CC01D1F1; **Taxon:** scientificNameID: *Occidozygashiwandashanensis*; scientificName: *Occidozygashiwandashanensis*; class: Amphibia; order: Anura; family: Dicroglossidae; genus: Occidozyga; specificEpithet: *shiwandashanensis*; scientificNameAuthorship: Chen, Peng, Liu, Huang, Liao and Mo, 2022; **Location:** country: Vietnam; countryCode: VN; stateProvince: Bac Giang; locality: Tay yen Tu Nature Reserve; verbatimElevation: 400 m; verbatimLatitude: 21°09.662’N; verbatimLongitude: 106°49.236’E; verbatimCoordinateSystem: WGS84; **Event:** eventDate: 22 May 2015; eventRemarks: collected by C. T. Pham; **Record Level:** language: en; collectionCode: Amphibia; basisOfRecord: PreservedSpecimen
- **Type status:** Other material. **Occurrence:** catalogNumber: IEBR A.5200; individualCount: 1; sex: male; lifeStage: adult; occurrenceID: D0631131-0D25-5669-8B67-6AD0E87FC1E3; **Taxon:** scientificNameID: *Occidozygashiwandashanensis*; scientificName: *Occidozygashiwandashanensis*; class: Amphibia; order: Anura; family: Dicroglossidae; genus: Occidozyga; specificEpithet: *shiwandashanensis*; scientificNameAuthorship: Chen, Peng, Liu, Huang, Liao and Mo, 2022; **Location:** country: Vietnam; countryCode: VN; stateProvince: Bac Giang; locality: Tay yen Tu Nature Reserve; verbatimElevation: 360 m; verbatimLatitude: 21°10.757’N; verbatimLongitude: 106°42.623’E; verbatimCoordinateSystem: WGS84; **Event:** eventDate: 12 June 2007; eventRemarks: collected by T. T. Tran; **Record Level:** language: en; collectionCode: Amphibia; basisOfRecord: PreservedSpecimen
- **Type status:** Other material. **Occurrence:** catalogNumber: IEBR A.5201; individualCount: 1; sex: female; lifeStage: adult; occurrenceID: C2F27082-CD49-52F9-B464-F6A442067019; **Taxon:** scientificNameID: *Occidozygashiwandashanensis*; scientificName: *Occidozygashiwandashanensis*; class: Amphibia; order: Anura; family: Dicroglossidae; genus: Occidozyga; specificEpithet: *shiwandashanensis*; scientificNameAuthorship: Chen, Peng, Liu, Huang, Liao and Mo, 2022; **Location:** country: Vietnam; countryCode: VN; stateProvince: Bac Giang; locality: Tay yen Tu Nature Reserve; verbatimElevation: 445 m; verbatimLatitude: 21°09.861’N; verbatimLongitude: 106°48.885’E; verbatimCoordinateSystem: WGS84; **Event:** eventDate: 16 June 2007; eventRemarks: collected by T. T. Tran; **Record Level:** language: en; collectionCode: Amphibia; basisOfRecord: PreservedSpecimen
- **Type status:** Other material. **Occurrence:** catalogNumber: IEBR A.5202; individualCount: 1; sex: female; lifeStage: adult; occurrenceID: A7BF3B31-E034-554B-A63D-644C6BDF4114; **Taxon:** scientificNameID: *Occidozygashiwandashanensis*; scientificName: *Occidozygashiwandashanensis*; class: Amphibia; order: Anura; family: Dicroglossidae; genus: Occidozyga; specificEpithet: *shiwandashanensis*; scientificNameAuthorship: Chen, Peng, Liu, Huang, Liao and Mo, 2022; **Location:** country: Vietnam; countryCode: VN; stateProvince: Bac Giang; locality: Tay yen Tu Nature Reserve; verbatimElevation: 330 m; verbatimLatitude: 21°10.131’N; verbatimLongitude: 106°48.727’E; verbatimCoordinateSystem: WGS84; **Event:** eventDate: 16 June 2007; eventRemarks: collected by T. T. Tran; **Record Level:** language: en; collectionCode: Amphibia; basisOfRecord: PreservedSpecimen
- **Type status:** Other material. **Occurrence:** catalogNumber: IEBR A.5203; individualCount: 1; sex: female; lifeStage: adult; occurrenceID: 3A267451-403C-5584-8EEA-CB325CB7EF4A; **Taxon:** scientificNameID: *Occidozygashiwandashanensis*; scientificName: *Occidozygashiwandashanensis*; class: Amphibia; order: Anura; family: Dicroglossidae; genus: Occidozyga; specificEpithet: *shiwandashanensis*; scientificNameAuthorship: Chen, Peng, Liu, Huang, Liao and Mo, 2022; **Location:** country: Vietnam; countryCode: VN; stateProvince: Bac Giang; locality: Tay yen Tu Nature Reserve; verbatimElevation: 341 m; verbatimLatitude: 21°09.796’N; verbatimLongitude: 106°49.293’E; verbatimCoordinateSystem: WGS84; **Event:** eventDate: 16 June 2007; eventRemarks: collected by T. T. Tran; **Record Level:** language: en; collectionCode: Amphibia; basisOfRecord: PreservedSpecimen

#### Description

Morphometrics of the specimens are provided in Suppl. material 4. Morphological characters of the specimens from Bac Giang Province agreed well with the original description of Chen et al. (2022). Size medium (SVL 28.1 mm in males, SVL 36.2–39.5 mm in females); head wider than long (HL/HW 0.86 in males, HL/HW 0.86–0.91 in females); snout round in dorsal and lateral views, projecting slightly over lower jaw; canthus rostralis broadly round; loreal region vertical and slightly concave; snout slightly shorter than eye diameter (ED/RL 1.06 in males, ED/RL 1.04–1.10 in females); internarial distance wider than interorbital distance and upper eyelid width (IND 2.7 mm, IOD 1.8 mm, UEW 2.5 mm in males; IND 2.9–3.6 mm, IOD 2.0–2.4 mm, UEW 2.3–2.8 mm in females); tympanum hidden; vomerine teeth absent; tongue fleshy, round posteriorly.

Fore-limbs robust, upper arm length shorter than forearm length (UAL/SVL 0.17, FAL/SVL 0.40 in males; UAL/SVL 0.15–0.16, FAL/SVL 0.37–0.39 in females); fingers free of webbing, relative finger lengths II< I < IV < III; tips of fingers pointed; dermal fringes absent; subarticular tubercles present, formula 1, 1, 2, 2; palmar tubercles prominent, round; inner and outer metatarsal present; nuptial pad on finger I present in male.

Hind-limbs short, thigh longer than tibia (FeL/SVL 0.43, TbL/SVL 0.41 in males; FeL/SVL 0.43–0.45, TbL/SVL 0.41–0.43 in females; tibia approximately 2.5 times longer than wide (TbL/TbW 2.48 in males, TbL/TbW 2.40–2.59 in females); tips of toes round, slightly expanded into disc; relative toe lengths I < II < V < III < IV; toes fully webbed; subarticular tubercles present, formula 1, 1, 2, 3, 2; inner metatarsal tubercle elongate; outer metatarsal tubercle absent; tibio-tarsal articulation reaching posterior edge of eye.

Skin: Dorsal surface shagreened with small, raised tubercles, more prominent and dense on tibia; distinctly raised supratympanic fold stretching from corner of eye to shoulder; dorsolateral fold absent; ventral surface of throat, chest, abdomen and thighs scattered with small glands.

**Figure 4.**
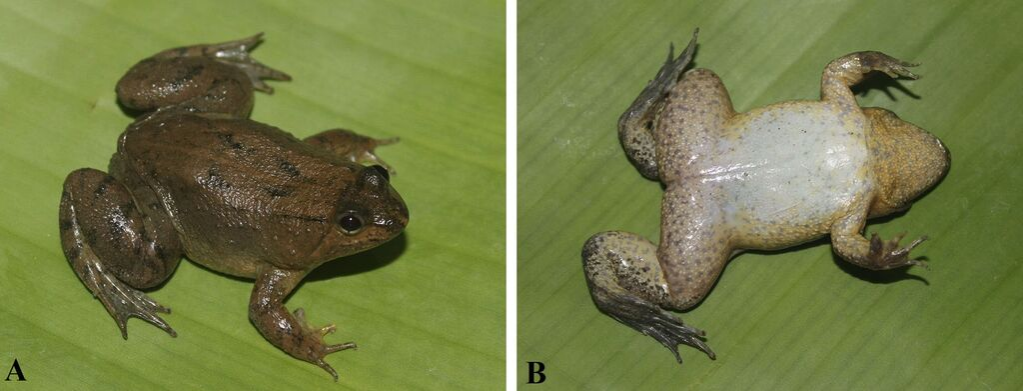
*Occidozygashiwandashanensis* (IEBR A.5199) from Bac Giang Province, Vietnam. **A** dorsolateral view; **B** ventral view.

Colouration in life: Dorsum pale brown with irregular pale dark spots with a light yellow vertebral stripe; dorsal surface of hind limbs pale brown with dark crossbars; ventral surface creamy-white with brown spots on lateral margin and throat; ventral surface of limbs yellow-white with dense brown spots; ventral surfaces of palm and feet brown; pupil reddish-brown; iris pale brown (Fig. 4) (determination after Chen et al. (2022)).

#### Distribution

The species was previously known only from the Shiwandashan Mountain, Fangcheng, Guangxi, China (Chen et al. 2022). The new record of this species in Bac Giang Province of Vietnam is approximately 180 km distant from the type locality in China.

#### Ecology

The specimens were found between 19:00 h and 23:00 h on the ground, in small ponds and in small streams. The surrounding habitat was mixed secondary evergreen forest consisting of larger and medium hardwoods, shrubs and arrowroot. The females contained yellowish-cream eggs with melanic poles. The specimens from Bac Giang Province were found at elevations of 300–400 m a.s.l., lower than the known altitude range in Guangxi, China (550–650 m a.s.l.) (Chen et al. 2022).

#### Notes

The specimen from Vietnam slightly differs from the type series from China by having the snout slightly shorter than eye diameter (vs. eye diameter less than snout length) and the presence of a light yellow vertebral stripe on the dorsum.

### Hylarana latouchii

(Boulenger, 1899)

C43B80C3-3104-5AC1-92B1-DFAB63B5A274

#### Materials

- **Type status:** Other material. **Occurrence:** catalogNumber: IEBR A.5204; individualCount: 1; sex: male; lifeStage: adult; occurrenceID: 3767F25E-2770-5CA8-A1D4-34DAF6C7254C; **Taxon:** scientificNameID: *Hylaranalatouchii*; scientificName: *Hylaranalatouchii*; class: Amphibia; order: Anura; family: Ranidae; genus: Hylarana; specificEpithet: *latouchii*; scientificNameAuthorship: Boulenger, 1899; **Location:** country: Vietnam; countryCode: VN; stateProvince: Hai Phong; locality: Cat Ba National Park; verbatimElevation: 78 m; verbatimLatitude: 20°48.142’N; verbatimLongitude: 101°01.486’E; verbatimCoordinateSystem: WGS84; **Event:** eventDate: 10 July 2020; eventRemarks: collected by T. Q. Phan and Q. H. Do; **Record Level:** language: en; collectionCode: Amphibia; basisOfRecord: PreservedSpecimen
- **Type status:** Other material. **Occurrence:** catalogNumber: IEBR A.5205; individualCount: 1; sex: male; lifeStage: adult; occurrenceID: B22337B1-3122-55CB-A68C-35805211EFCC; **Taxon:** scientificNameID: *Hylaranalatouchii*; scientificName: *Hylaranalatouchii*; class: Amphibia; order: Anura; family: Ranidae; genus: Hylarana; specificEpithet: *latouchii*; scientificNameAuthorship: Boulenger, 1899; **Location:** country: Vietnam; countryCode: VN; stateProvince: Quang Ninh; locality: Bai Tu Long National Park; verbatimElevation: 28 m; verbatimLatitude: 21°07.917’N; verbatimLongitude: 107°66.306’E; verbatimCoordinateSystem: WGS84; **Event:** eventDate: 26 June 2017; eventRemarks: collected by C. T. Pham; **Record Level:** language: en; collectionCode: Amphibia; basisOfRecord: PreservedSpecimen
- **Type status:** Other material. **Occurrence:** catalogNumber: IEBR A.5206; individualCount: 1; sex: male; lifeStage: adult; occurrenceID: 3C16C06B-7DD0-5840-987F-F3ABB35052CC; **Taxon:** scientificNameID: *Hylaranalatouchii*; scientificName: *Hylaranalatouchii*; class: Amphibia; order: Anura; family: Ranidae; genus: Hylarana; specificEpithet: *latouchii*; scientificNameAuthorship: Boulenger, 1899; **Location:** country: Vietnam; countryCode: VN; stateProvince: Quang Ninh; locality: Bai Tu Long National Park; verbatimElevation: 36 m; verbatimLatitude: 21°07.825’N; verbatimLongitude: 107°66.298’E; verbatimCoordinateSystem: WGS84; **Event:** eventDate: 28 June 2017; eventRemarks: collected by C. T. Pham; **Record Level:** language: en; collectionCode: Amphibia; basisOfRecord: PreservedSpecimen
- **Type status:** Other material. **Occurrence:** catalogNumber: IEBR A.2013.27; individualCount: 1; sex: male; lifeStage: adult; occurrenceID: 4C87555C-868F-580E-9034-96A2CC91845E; **Taxon:** scientificNameID: *Hylaranalatouchii*; scientificName: *Hylaranalatouchii*; class: Amphibia; order: Anura; family: Ranidae; genus: Hylarana; specificEpithet: *latouchii*; scientificNameAuthorship: Boulenger, 1899; **Location:** country: Vietnam; countryCode: VN; stateProvince: Quang Ninh; locality: Bai Tu Long National Park; verbatimElevation: 45 m; verbatimLatitude: 21°24.944’N; verbatimLongitude: 107°75.861’E; verbatimCoordinateSystem: WGS84; **Event:** eventDate: 15 May 2011; eventRemarks: collected by C. T. Pham, T. Ziegler, A. Gawor, and A. Dogra; **Record Level:** language: en; collectionCode: Amphibia; basisOfRecord: PreservedSpecimen
- **Type status:** Other material. **Occurrence:** catalogNumber: IEBR A.2013.29; individualCount: 1; sex: male; lifeStage: adult; occurrenceID: F68198B6-79F6-5E54-8BE8-9EE2DE14CEE4; **Taxon:** scientificNameID: *Hylaranalatouchii*; scientificName: *Hylaranalatouchii*; class: Amphibia; order: Anura; family: Ranidae; genus: Hylarana; specificEpithet: *latouchii*; scientificNameAuthorship: Boulenger, 1899; **Location:** country: Vietnam; countryCode: VN; stateProvince: Quang Ninh; locality: Bai Tu Long National Park; verbatimElevation: 65 m; verbatimLatitude: 21°24.823’N; verbatimLongitude: 107°75.781’E; verbatimCoordinateSystem: WGS84; **Event:** eventDate: 16 May 2011; eventRemarks: collected by C. T. Pham, T. Ziegler, A. Gawor, and A. Dogra; **Record Level:** language: en; collectionCode: Amphibia; basisOfRecord: PreservedSpecimen
- **Type status:** Other material. **Occurrence:** catalogNumber: IEBR A.2013.30; individualCount: 1; sex: male; lifeStage: adult; occurrenceID: AB6F2BAE-8099-50E5-8740-25652A660A36; **Taxon:** scientificNameID: *Hylaranalatouchii*; scientificName: *Hylaranalatouchii*; class: Amphibia; order: Anura; family: Ranidae; genus: Hylarana; specificEpithet: *latouchii*; scientificNameAuthorship: Boulenger, 1899; **Location:** country: Vietnam; countryCode: VN; stateProvince: Quang Ninh; locality: Bai Tu Long National Park; verbatimElevation: 65 m; verbatimLatitude: 21°24.823’N; verbatimLongitude: 107°75.781’E; verbatimCoordinateSystem: WGS84; **Event:** eventDate: 16 May 2011; eventRemarks: collected by C. T. Pham, T. Ziegler, A. Gawor, and A. Dogra; **Record Level:** language: en; collectionCode: Amphibia; basisOfRecord: PreservedSpecimen
- **Type status:** Other material. **Occurrence:** catalogNumber: IEBR A.2013.41; individualCount: 1; sex: female; lifeStage: adult; occurrenceID: 2941BE01-FAF8-5388-A8BE-122AE5DED862; **Taxon:** scientificNameID: *Hylaranalatouchii*; scientificName: *Hylaranalatouchii*; class: Amphibia; order: Anura; family: Ranidae; genus: Hylarana; specificEpithet: *latouchii*; scientificNameAuthorship: Boulenger, 1899; **Location:** country: Vietnam; countryCode: VN; stateProvince: Quang Ninh; locality: Bai Tu Long National Park; verbatimElevation: 12 m; verbatimLatitude: 21°18.139’N; verbatimLongitude: 107°66.694’E; verbatimCoordinateSystem: WGS84; **Event:** eventDate: 18 May 2011; eventRemarks: collected by C. T. Pham, T. Ziegler, A. Gawor, and A. Dogra; **Record Level:** language: en; collectionCode: Amphibia; basisOfRecord: PreservedSpecimen
- **Type status:** Other material. **Occurrence:** catalogNumber: IEBR A.5204; individualCount: 1; sex: male; lifeStage: adult; occurrenceID: 5F344AF9-EC5A-5C74-B74C-8E9C01F70215; **Taxon:** scientificNameID: *Hylaranalatouchii*; scientificName: *Hylaranalatouchii*; class: Amphibia; order: Anura; family: Ranidae; genus: Hylarana; specificEpithet: *latouchii*; scientificNameAuthorship: Boulenger, 1899; **Location:** country: Vietnam; countryCode: VN; stateProvince: Hai Phong; locality: Cat Ba National Park; verbatimElevation: 78 m; verbatimLatitude: 20°48.142’N; verbatimLongitude: 101°01.486’E; verbatimCoordinateSystem: WGS84; **Event:** eventDate: 10 July 2020; eventRemarks: collected by T. Q. Phan and Q. H. Do; **Record Level:** language: en; collectionCode: Amphibia; basisOfRecord: PreservedSpecimen

#### Description

Morphometrics of the specimens are provided in Suppl. material 5. Morphological characters of the specimens from Hai Phong City and Quang Ninh Province agreed well with the descriptions of Fei et al. (2009) and Fei et al. (2012). Size medium (SVL 48.6–51.7 mm in males, SVL 58.4 mm in females); head longer than wide (HL/HW 1.10–1.13 in males, HL/HW 1.17 in females); snout round in dorsal view, projecting beyond lower jaw; nostril lateral, closer to tip of snout than to eye (NS/EN 0.74–0.91 in males, NS/EN 0.80 in females); canthus rostralis distinct; pupil horizontally oval; loreal region slightly concave and oblique; snout length greater than eye diameter (ED/RL 0.85–0.92 in males, ED/RL 0.83 in females); internarial distance wider than interorbital distance and upper eyelid (IND 5.2–5.6 mm, IOD 4.6–5.1 mm, UEW 3.8–4.2 mm in males; IND 6.7 mm, IOD 5.3 mm, UEW 4.8 mm in females); tympanum distinct, round, smaller than eye diameter (TD/ED 0.60–0.68 in males, TD/ED 0.64 in females); vomerine teeth present, in two oblique ridges; tongue cordiform, deeply notched posteriorly.

Fore-limbs robust, upper arm length shorter than forearm length (UAL/SVL 0.21–23, FAL/SVL 0.44–0.49 in males; UAL/SVL 0.22, FAL/SVL 0.47 in females); fingers free of webbing, relative finger lengths II < I < IV < III; tips of fingers round, not expanded into disc; subarticular tubercles present, formula 1, 1, 2, 2; palmar tubercles prominent, round; inner and outer metatarsal present; nuptial pad on finger I present in males.

Hind-limbs short, thigh longer than tibia (FeL/SVL 0.47–0.49, TbL/SVL 0.51–0.55 in males; FeL/SVL 0.47, TbL/SVL 0.51 in females; tibia approximately five times longer than wide (TbL/TbW 4.44-5.06 in males, TbL/TbW 5.03 in females); tips of toes round, slightly expanded into disc; relative toe lengths I < II < V < III < IV; webbing well developed, formula I1/2–1II1/3–11/2III1/2–2V11/2–0V; subarticular tubercles present, formula 1, 1, 2, 3, 2; inner metatarsal tubercle elongate; outer metatarsal tubercle small and round; tibio-tarsal articulation reaching to between eye and nostril.

Skin: Dorsal surface shagreened with tubercles, more prominent on posterior of dorsum and flank; tiny spinules on upper edge of eyelid, anterior and posterior edge of tympanum; supratympanic fold absent; dorsolateral fold present; dorsal surface of fore-limbs smooth; throat, chest, belly and ventral surface of thigh smooth.

**Figure 5.**
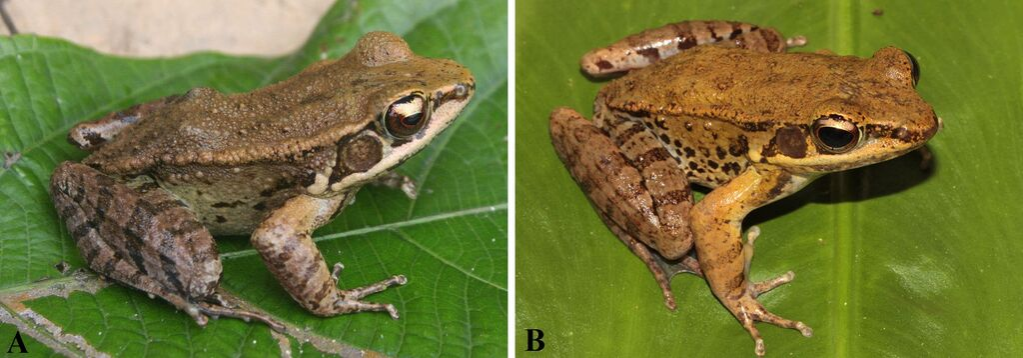
*Hylaranalatouchii*. **A** male (IEBR A.5204) from Quang Ninh Province, Vietnam; **B** male (IEBR A.5205) from Hai Phong City, Vietnam.

Colouration in life: Iris black, surrounded by red-golden network; dorsum light yellow or grey yellow; flanks yellowish-white or with dark spots; dorsal surface of fore- and hind-limbs brown with dark brown cross bands; upper lip white; throat, chest, belly and ventral surface of thigh cream with dark brown mottling (Fig. 5) (determination after Fei et al. (2009) and Fei et al. (2012)).

#### Distribution

The species was previously known only from southern China (Zhejiang, Fujian, Guangxi, Hong Kong, Guangdong, Hunan, Jiangxi, Jiangxu and Anhui), including Taiwan (Frost 2023). The new record of this species in Hai Phong City and Quang Ninh Province, Vietnam is approximately 1,300 km distant from the type locality in Fuzhou, Fujian Province, China.

#### Ecology

The specimens were found between 19:00 h and 23:00 h on the ground, in small ponds and in small rocky streams. The surrounding habitat was mixed secondary karst forest and evergreen forest of medium hardwoods, shrubs and arrowroot.

#### Notes

The specimens from Vietnam slightly differ from the type series from China by having a slightly larger size (SVL 48.6-51.7 mm in males, SVL 58.4 mm in females vs. 36.0-40.0 mm in males, 42.0-53.0 in females).

## Discussion

In their herpetofaunal list of Vietnam, Nguyen et al. (2009) listed three species of the genus *Occidozyga* from the country (*O.lima*, *O.martensii* and *O.vittata*). Poyarkov et al. (2020), based on morphological concordance, considered Oxyglossuslaevisvar.vittata Andersson, 1942 (*Occidozygavittata*) to be a junior synonym of *Occidozygamartensii*. The *O.martensii* group represents a species complex (Lyu et al. 2022) and *O.lingnanica* Lyu and Wang, 2022, currently described from south-eastern China, is one species of the *O.martensii* complex. Therefore, it is necessary to study carefully both the morphology and the molecular biology of *Occidozyga* from Vietnam.

In the genus *Hylarana*, 14 species have been known from Vietnam, namely *Hylaranaannamitica* Sheridan & Stuart, 2018; *H.attigua* (Inger, Orlov & Darevsky, 1999); *H.cubitalis* (Smith, 1917); *H.glandulosa* (Boulenger, 1882); *H.guentheri* (Boulenger, 1882); *H.erythraea* (Schlegel, 1837); *H.lateralis* (Boulenger, 1887); *H.macrodactyla* Günther, 1858; *H.maosonensis* (Bourret, 1937); *H.milleti* (Smith, 1921); *H.montivaga* (Smith, 1921); *H.montosa* Sheridan & Stuart, 2018; *H.nigrovittata* (Blyth, 1856); and *H.taipehensis* (Van Denburgh, 1909) (Frost 2023).

The new country record of *Hylaranalatouchii* from Vietnam, which was already mentioned by Gawor et al. (2016), however, without specific identification “the taxonomic status of the *Hylarana* from Bai Tu Long needs further clarification”, brings the total number of *Hylarana* to 15 in Vietnam.

Our research also showed that the genus *Hylarana* contains several species complexes. Interspecific genetic divergences of the species complexes is relatively high, for example, between population of *H.latouchii*, these were up to 2.6%, but still lower than those of *H.maosonensis* (up to 4.44%), *H.annamitica* (3.55%) and *H.nigrovittata* (2.8%).

These new discoveries highlight that the knowledge on the herpetofauna of Vietnam, particularly in the border region between China and Vietnam, is still incomplete and that additional field research is warranted.

## Supplementary Material

XML Treatment for Occidozyga shiwandashanensis

XML Treatment for Hylarana latouchii

F5387D7D-8054-5CE8-A731-EF7CD2C86598 10.3897/BDJ.11.e109726.suppl1 Supplementary material 1 GenBank accession numbers and associated samples Data type Sampling information Brief description Localities, voucher ID and GenBank numbers for all samples used in this study. File: oo_920145.docx https://binary.pensoft.net/file/920145 Tung Thanh Tran, Chung Van Hoang, Anh Mai Luong, Truong Quang Nguyen, Thomas Ziegler, Cuong The Pham

DD97FDFD-8A74-576C-B958-1A9949EE4D7F 10.3897/BDJ.11.e109726.suppl2 Supplementary material 2 Uncorrected (“p”) distance matrix of 11 species of *Occidozyga* Data type Genetic divergence Brief description Uncorrected (“p”) distance matrix showing percentage pairwise genetic divergence 16S between the sequence of collected sample and available sequences of 11 species of *Occidozyga* in GenBank. File: oo_879306.docx https://binary.pensoft.net/file/879306 Tung Thanh Tran, Chung Van Hoang, Anh Mai Luong, Truong Quang Nguyen, Thomas Ziegler, Cuong The Pham

02744EF6-EADF-5E51-A03E-D38FF126BE55 10.3897/BDJ.11.e109726.suppl3 Supplementary material 3 Uncorrected (“p”) distance matrix of seven species of *Hylarana* Data type Genetic divergence Brief description Uncorrected (“p”) distance matrix showing percentage pairwise genetic divergence 16S between the two sequences of collected samples and available sequences of seven species of *Hylarana* in GenBank. File: oo_879308.docx https://binary.pensoft.net/file/879308 Tung Thanh Tran, Chung Van Hoang, Anh Mai Luong, Truong Quang Nguyen, Thomas Ziegler, Cuong The Pham

2BC2DEEB-1BD4-5647-AE8F-A03F04FBD919 10.3897/BDJ.11.e109726.suppl4 Supplementary material 4 Measurements of *Occidozygashiwandashanensis* Data type Mophological Data Brief description Measurement (in mm) and proportions of *Occidozygashiwandashanensis*. File: oo_911840.docx https://binary.pensoft.net/file/911840 Tung Thanh Tran, Chung Van Hoang, Anh Mai Luong, Truong Quang Nguyen, Thomas Ziegler and Cuong The Pham

5321E6FB-97CB-5F56-9E1A-F201DF5F90CB 10.3897/BDJ.11.e109726.suppl5 Supplementary material 5 Measurements of *Hylaranalatouchii* Data type Morphological data Brief description Measurements (in mm) and proportions of *Hylaranalatouchii*. File: oo_877865.docx https://binary.pensoft.net/file/877865 Tung Thanh Tran, Chung Van Hoang, Anh Mai Luong, Truong Quang Nguyen, Thomas Ziegler, Cuong The Pham

## References

[B10040612] Chen W. C., Peng W., Liu Y., Huang Z., Liao X. W., Mo Y. M. (2022). A new species of *Occidozyga* Kuhl and van Hasselt, 1822 (Anura: Dicroglossidae) from Southern Guangxi, China. Zoological Research.

[B10040631] Fei L., Hu S. Q., Ye C. Y., Huang Y. Z. (2009). Fauna Sinica. Amphibia. Vol. 3. Anura.

[B10040639] Fei L., Ye C. Y., Jiang J. P. (2012). Colored atlas of Chinese amphibians and their distributions.

[B10040647] Frost DR Amphibian Species of the World. http://research.amnh.org/herpetology/amphibia/index.html.

[B10040655] Gawor A., Pham C. T, Nguyen T. Q., Nguyen T. T., Schmitz A, Ziegler T. (2016). The herpetofauna of the Bai Tu Long National Park, northeast Vietnam. Salamandra.

[B10040666] Glaw F., Vences M. (2007). A field guide to the Amphibians and Reptiles of Madagascar.

[B10040674] Hall T. A. (1999). BioEdit: a user-friendly biological sequence alignment editor and analysis program for Windows 95/98/NT. Nucleic Acids Symposium Series.

[B10040683] Hillis D. M., Bull J. J. (1993). An empirical test of bootstrapping as a method for assessing confidence in phylogenetic analysis. Systematic Biology.

[B10040692] Hoang C. V., Nguyen T. T., Phan T. Q., Pham C. T., Ninh H. T., Wang B., Jiang J., Ziegler T., Nguyen T. Q. (2022). Distribution pattern of the *Microhylaheymonsi* group (Anura, Microhylidae) with descriptions of two new species from Vietnam. European Journal of Taxonomy.

[B10040706] Huelsenbeck J. P., Hillis D. M. (1993). Success of Phylogenetic Methods in the Four-Taxon Case. Systematic Biology.

[B10040715] Kumar S., Stecher G., Tamura K. (2016). MEGA7: Molecular Evolutionary Genetics Analysis version 7.0 for bigger datasets. Molecular Biology and Evolution.

[B10040724] Leaché A. D., Reeder T. W. (2002). Molecular systematics of the eastern fence lizard (*Sceloporusundulatus*): a comparison of parsimony, likelihood, and Bayesian approaches. Systematic Biology.

[B10040733] Liu S., Mo M., Rao D. (2022). First country record of the fanged frog *Limnonectesnguyenorum* McLeod, Kurlbaum & Hoang, 2015 (Anura, Dicroglossidae) in China. Herpetozoa.

[B10040742] Lyu Z. T., Wang J., Zeng Z. C., Luo L., Zhang Y. W., Guo C. P., Ren J. L., Qi S., Mo Y. M., Wang Y. Y. (2022). Taxonomic clarifications on the floating frogs (Anura: Dicroglossidae: *Occidozyga* sensu lato) in southeastern China. Vertebrate Zoology.

[B10040766] Matsui M., Ohler A., Eto K., Nguyen T. T. (2017). Distinction of *Gracixaluscarinensis* from Vietnam and Myanmar, with description of a new species. Alytes.

[B10040757] McLeod D. S., Kurlbaum S., Hoang N. V. (2015). More of the same: a diminutive new species of the *Limnonecteskuhlii* complex from northern Vietnam (Anura: Dicroglossidae). Zootaxa.

[B10040775] Mo Y., Chen W., Wu H., Zhang W., Zhou S. (2015). A new species of *Odorrana* inhabiting complete darkness in a karst cave in Guangxi, China. Asian Herpetological Research.

[B10040785] Nguyen L. T., Tapley B., Nguyen C. T., Luong H. V., Rowley J. J.L. (2021). A new species of *Leptobrachella* (Anura, Megophryidae) from Mount Pu Ta Leng, northwest Vietnam. Zootaxa.

[B10040795] Nguyen T. Q., Le M. D., Pham C. T., Nguyen T. T., Bonkowski M., Ziegler T. (2013). A new species of *Gracixalus* (Amphibia: Anura: Rhacophoridae) from northern Vietnam. Organisms Diversity & Evolution.

[B10041399] Nguyen V. S., Ho T. C., Nguyen T. Q. (2009). Herpetofauna of Vietnam.

[B10040820] Ninh H. T., Nguyen T. T., Orlov N. L., Nguyen T. Q., Ziegler T. (2020). A new species of the genus *Zhangixalus* (Amphibia: Rhacophoridae) from Vietnam. European Journal of Taxonomy.

[B10040806] Ninh H. T., Nguyen T. T., Nguyen H. Q., Hoang N. V., Siliyavong S., Nguyen T. V., Le D. T., Le Q. K., Ziegler T. (2022). A new species of mossy frog (Anura: Rhacophoridae) from Northeastern Vietnam. European Journal of Taxonomy.

[B10040830] Nishikawa K., Matsui M., Nguyen T. T. (2013). A New Species of *Tylototriton* from northern Vietnam (Amphibia: Urodela: Salamandridae). Current Herpetology.

[B10040870] Pham C. T., Nguyen T. Q., Bernardes M., Nguyen T. T., Ziegler T. (2016). First records of *Bufogargarizans* Cantor, 1842 and *Odorranalipuensis* Mo, Chen, Wu, Zhang et Zhou, 2015 (Anura: Bufonidae) from Vietnam. Russian Journal of Herpetology.

[B10040880] Pham C. T., Nguyen T. Q., Le M. D., Bonkowski M., Ziegler T. (2016). A new species of *Odorrana* (Amphibia: Anura: Ranidae) from Vietnam. Zootaxa.

[B10040839] Pham C. T., Le M. D., Nguyen T. T., Ziegler T., Wu Z. J., Nguyen T. Q. (2017). A new species of *Limnonectes* (Amphibia: Anura: Dicroglossidae) from Vietnam. Zootaxa.

[B10040861] Pham C. T., Le M. D., Ngo H. T., Nguyen T. Q. (2020). New records of Cascade Frogs of the genus *Odorrana* (Amphibia: Anura: Ranidae) from Vietnam. Academia Journal of Biology.

[B10040850] Pham C. T., Le M. D., Hoang C. V., Pham A. V., Ziegler T., Nguye T. Q. (2020). First records of *Bufoluchunnicus* (Yang et Rao, 2008) and *Amolopswenshanensis* Yuan, Jin, Li, Stuart et Wu, 2018 (Anura: Bufonidae, Ranidae) from Vietnam. Russian Journal of Herpetology.

[B10040890] Poyarkov N. A., Solovyeva E. V., Nguyen T. V., Geissler P. (2020). On the taxonomic status of three enigmatic Indochinese frog species (Amphibia: Anura) described by L. G. Andersson. Zootaxa.

[B10040899] Rambaut A., Drummond A. TRACER, version 1.5.. http://beast.bio.ed.ac.uk/Tracer.

[B10040907] Ronquist F., Teslenko M., Mark P., Ayres D. L., Darling A., Höhna S., Larget B., Liu L., Suchard M. A., Huelsenbeck J. P. (2012). MrBayes 3.2: efficient Bayesian phylogenetic inference and model choice across a large model space. Systematic Biology.

[B10040922] Simmons J. E. (2002). Herpetological collecting and collections management. Revised edition. Society for the Study of Amphibians and Reptiles. Herpetological Circular.

[B10040931] Simon C., Frati F., Beckenbach A., Crespi B., Liu H., Flook P. (1994). Evolution, weighting, and phylogenetic utility of mitochondrial gene sequences and a compilation of conserved polymerase chain reaction primers. Annals of the Entomological Society of America.

[B10502217] Sterling E. J., Hurley M. M., Le D. M. (2006). Vietnam: A Natural History.

[B10040942] Sumida M., Ueda H,, Nishioka M. (2003). Reproductive isolating mechanisms and molecular phylogenetic relationships among Palearctic and Oriental brown frogs. Zoological Science.

[B10040951] Sun Y. J., Zheng Y. Y., Zheng W. C., Lin Z. H., Qiao F. (2021). The complete mitochondrial genome of *Hylaranalatouchii* (Anura: Ranidae) and its phylogenetic analysis. Mitochondrial DNA Part B.

[B10040961] Tanabe A. S. (2011). Kakusan 4 and Aminosan: two programs for comparing nonpartitioned, proportional and separate models for combined molecular phylogenetic analyses of multilocus sequence data. Molecular Ecology Resources.

[B10040970] Tapley B., Cutajar T. P., Mahony S., Nguyen C. T., Dau Q. V., Luong A. M., Le D. T., Nguyen T. T., Nguyen T. Q., Portway C., Luong H. V., Rowley J. J.L. (2018). Two new and potentially highly threatened *Megophrys* Horned frogs (Amphibia: Megophryidae) from Indochina’s highest mountains. Zootaxa.

[B10040987] Thompson J. D., Gibson T. J., Plewniak F., Jeanmougin F., Higgins D. G. (1997). The CLUSTAL_X windows interface: flexible strategies for multiple sequence alignment aided by quality analysis tools. Nucleic Acids Research.

[B10040997] Tran T. T., Pham A. V., Le M. D., Nguyen N. H., Ziegler T., Pham C. T. (2023). A new species of *Gracixalus* (Anura, Rhacophoridae) from northwestern Vietnam. ZooKeys.

[B10041008] Wang Y., Lau M. W., Yang J., Chen G., Liu Z., Pang H., Liu Y. (2015). A new species of the genus *Odorrana* (Amphibia: Ranidae) and the first record of *Odorranabacboensis* from China. Zootaxa.

[B10041020] Wu Y. H., Yu Z. B., Lu C. Q., Felista K. K., Hou S., Jin J., Chen J. M., Zhang D., Che J. (2023). First national record of *Microhylahmongorum* Hoang, Nguyen, Phan, Pham, Ninh, Wang, Jiang, Ziegler and Nguyen, 2022 (Anura, Microhylidae, Microhyla) in China. Biodiversity Data Journal.

[B10041039] Xiao Y., Xia Y., Zeng X. (2019). The mitochondrial genome of broad-folded frog (*Hylaranalatouthii*). Mitochondrial DNA Part B.

[B10041048] Ye J., Wei Z., Han F., Ni Q., Yao Y., Xu H., Li Y., Rao D., Zhang M. (2017). The complete mitogenome sequence of *Tylototritonziegleri* (Amphibia: Caudata). Conservation Genetics Resources.

[B10041062] Yuan Z., Jin J., Li J., Stuart B. L., Wu J. (2018). A new species of cascade frog (Amphibia: Ranidae) in the *Amolopsmonticola* group from China. Zootaxa.

