# Supplementary material for: Integrative taxonomic analyses reveal first country records of Occidozygashiwandashanensis Chen, Peng, Liu, Huang, Liao & Mo, 2022 and Hylaranalatouchii (Boulenger, 1899) (Anura, Dicroglossidae, Ranidae) from Vietnam
Source: Biodivers Data J. 2023 Oct 13;11:e109726. doi: 10.3897/BDJ.11.e109726 (PMC10589760; doi:10.3897/BDJ.11.e109726)
Supplement: Supplementary material 1 — GenBank accession numbers and associated samples [file bdj-11-e109726-s001.docx]

**Table S1: GenBank accession numbers and associated samples that were used in this study**

|  | **Species** | **No Genbank** | **Voucher** | **Location** | **References** |
| --- | --- | --- | --- | --- | --- |
| **1** | *O. shiwandashanensis* OR656682 | | IEBR A.5199 | Bac Giang, Vietnam | This study |
| **2** | *O. shiwandashanensis* | MZ747458 | NNU202103321 | Guangxi, China | Chen et al. 2022 |
| **3** | *O. shiwandashanensis* | MZ747456 | NNU202103285 | Guangxi, China | Chen et al. 2022 |
| **4** | *O. shiwandashanensis* | MZ747457 | NNU202103320 | Guangxi, China | Chen et al. 2022 |
| **5** | *O. shiwandashanensis* | MZ747455 | NNU202103284 | Guangxi, China | Chen et al. 2022 |
| **6** | *O. berbeza* | LC593607 | KUHE 17327 | Matang, Sarawak | Matsui et al. 2021, Chen et al. 2022 |
| **7** | *O. laevis* | AY313684 | PNM 7446 | Luzon, Philippines | Evans et al. 2003, Chen et al. 2022 |
| **8** | *O. sumatrana* | LC593611 | MZB Amph 16392 | Java | Matsui et al. 2021, Chen et al. 2022 |
| **9** | *O. baluensis* | DQ283143 | FMNH 242747 | Sipitang, Sabah | Frost et al. 2006, Chen et al. 2022 |
| **10** | *O. diminutiva* | MT820199 | KU 321225 | Pasonanca, Philippines | Chan et al. 2021, Chen et al. 2022 |
| **11** | *O. lima* | DQ283224 | CAS 213254 | Yangon, Myanmar | Frost et al. 2006, Chen et al. 2022 |
| **12** | *O. magnapustulosa* | MW217488 | GK_7395 | Thailand | Köhler et al. 2021, Chen et al. 2022 |
| **13** | *O. myanhesei* | MW217501 | SMF 103797 | *Thanlyin, Yangon, Myanmar | Köhler et al. 2021, Chen et al. 2022 |
| **14** | *O. martensii* | DQ283357 | AMNH A161171 | HaTinh, Vietnam | Frost et al. 2006, Chen et al. 2022 |
| **15** | *O. swanbornorum* | MN705433 | JnUZool-A0719 | Chattogram, Bangladesh | Trageser et al. 2021, Chen et al. 2022 |
| **Outgroup** | *Limnonectes jarujini* | AB558944 | KUHE 20127 | Thailand | Matsui et al. 2010, Chen et al. 2022 |
| **Outgroup** | *Ingerana tenasserimensis* | AY322302 | CAS 205064 | Myanmar | Roelants et al. 2004, Chen et al. 2022 |
| **16** | *H. latouchii* | OR656680 | IEBR A.5205 | Quang Ninh, Vietnam | This study |
| **17** | *H. latouchii* | OR656681 | IEBR A.5204 | Hai Phong, Vietnam | This study |
| **18** | *H. latouchii* | MT702387 | LSU20200422001ZL | Jiangxi, China | Zheng et al. 2020 |
| **19** | *H. latouchii* | MN241431 | NT | Zhejiang, China | Xiao et al. 2019 |
| **20** | *H. latouchii* | AB058880 | NT | Taiwan, China | Sumida et al. 2003 |
| **21** | *H. latouchii* | LC640538 | NT | NT | Kambayashi et al. 2022 |
| **22** | *H. spinulosa* | KU840599 | NT | NT | Goutte et al. 2016 |
| **23** | *H. spinulosa* | KR264109 | ROM44390 | NT | Oliver et al. 2015 |
| **24** | *H. spinulosa* | KR264093 | MVZ236683 | NT | Oliver et al. 2015 |
| **25** | *H. spinulosa* | KF185067 | HNNU 051117 | Hainan, China | Chen et al. 2013 |
| **26** | *H. spinulosa* | DQ360004 | SCUMH010 | NT | Che et al. 2007 |
| **27** | *H. maosonensis* | DQ283373 | AMNH A161487 | Vinh Phu, Vietnam | Frost et al. 2006 |
| **28** | *H. maosonensis* | OM387171 | USNM:Herp:595524 | Ha Giang, Vietnam | Miller et al. 2022 |
| **29** | *H. maosonensis* | EU754852 | ZFMK 87295 | Quang Binh, Vietnam | Gawor et al. 2009 |
| **30** | *H. maosonensis* | AF206488 | ROM 24274 | Hai Duong, Vietnam | Chen et al. 2005 |
| **31** | *H. maosonensis* | OM387187 | USNM:Herp:595525 | Ha Giang, Vietnam | Miller et al. 2022 |
| **32** | *H. maosonensis* | KR264072 | FMNH255637 | NT | Oliver et al. 2015 |
| **33** | *H. maosonensis* | KU840600 | NT | NT | Goutte et al. 2016 |
| **34** | *H. maosonensis* | HQ337893 | NT | NT | Gawor et al. 2010 |
| **35** | *H. cubitalis* | KR264077 | FMNH265818 | NT | Oliver et al. 2015 |
| **36** | *H. lacrima* | MW076232 | MZUHC 465 | Mizoram, India | Lalronunga et al. 2020 |
| **37** | *H. lacrima* | MW076230 | MZUHC 459 | Mizoram, India | Lalronunga et al. 2020 |
| **38** | *H. lacrima* | MW076231 | MZUHC 464 | Mizoram, India | Lalronunga et al. 2020 |
| **39** | *H. lacrima* | MG935996 | USNM:Herp:583126 | Mandalay, Myanmar | Mulcahy et al. 2018 |
| **40** | *H. annamitica* | KU840602 | NT | NT | Goutte et al. 2016 |
| **41** | *H. annamitica* | DQ283371 | AMNH A161280 | Ha Tinh, Vietnam | Frost et al. 2006 |
| **42** | *H. nigrovittata* | KR264114 | USNM583178 | NT | Oliver et al. 2015 |
| **43** | *H. nigrovittata* | MG935999 | USNM:Herp:583178 | Mon, Myanmar | Mulcahy et al. 2018 |
| **44** | *H. nigrovittata* | AY322277 | NT | NT | Roelants et al. 2004 |
| **45** | *H. nigrovittata* | AB719238 | KUHE:23726 | Chiang Dao, Thailand | Matsui et al. 2012 |
| **46** | *H. nigrovittata* | MG936000 | USNM:Herp:583174 | Mandalay, Myanmar | Mulcahy et al. 2018 |
| **Outgroup** | *Bambina holsti* | AB761264 | NT | Okinawa, Japan | Kurabayashi et al. 2012 |
