# Supplementary material for: Integrative taxonomic analyses reveal first country records of Occidozygashiwandashanensis Chen, Peng, Liu, Huang, Liao & Mo, 2022 and Hylaranalatouchii (Boulenger, 1899) (Anura, Dicroglossidae, Ranidae) from Vietnam
Source: Biodivers Data J. 2023 Oct 13;11:e109726. doi: 10.3897/BDJ.11.e109726 (PMC10589760; doi:10.3897/BDJ.11.e109726)
Supplement: Supplementary material 2 — Uncorrected (“p”) distance matrix of 11 species of Occidozyga [file bdj-11-e109726-s002.docx]

**Table S2: Uncorrected (“p”) distance matrix showing percentage pairwise genetic divergence 16S between the sequence of collected sample and available sequences of 11 species of *Occidoziga* in GenBank.**

|  | **Species** | **1** | **2** | **3** | **4** | **5** | **6** | **7** | **8** | **9** | **10** | **11** | **12** | **13** | **14** | **15** |
| --- | --- | --- | --- | --- | --- | --- | --- | --- | --- | --- | --- | --- | --- | --- | --- | --- |
| **1** | *O. shiwandashanensis* IEBR A.5199 |  |  |  |  |  |  |  |  |  |  |  |  |  |  |  |
| **2** | *O. shiwandashanensis* MZ747458 | 0.000 |  |  |  |  |  |  |  |  |  |  |  |  |  |  |
| **3** | *O. shiwandashanensis* MZ747456 | 0.015 | 0.015 |  |  |  |  |  |  |  |  |  |  |  |  |  |
| **4** | *O. shiwandashanensis* MZ747457 | 0.000 | 0.000 | 0.015 |  |  |  |  |  |  |  |  |  |  |  |  |
| **5** | *O. shiwandashanensis* MZ747455 | 0.015 | 0.015 | 0.000 | 0.015 |  |  |  |  |  |  |  |  |  |  |  |
| **6** | *O. berbeza* | 0.145 | 0.145 | 0.143 | 0.145 | 0.143 |  |  |  |  |  |  |  |  |  |  |
| **7** | *O. laevis* | 0.143 | 0.148 | 0.137 | 0.148 | 0.137 | 0.184 |  |  |  |  |  |  |  |  |  |
| **8** | *O. sumatrana* | 0.136 | 0.142 | 0.133 | 0.142 | 0.133 | 0.166 | 0.129 |  |  |  |  |  |  |  |  |
| **9** | *O. baluensis* | 0.145 | 0.152 | 0.148 | 0.152 | 0.148 | 0.200 | 0.176 | 0.149 |  |  |  |  |  |  |  |
| **10** | *O. diminutiva* | 0.147 | 0.147 | 0.147 | 0.147 | 0.147 | 0.167 | 0.169 | 0.151 | 0.099 |  |  |  |  |  |  |
| **11** | *O. lima* | 0.149 | 0.156 | 0.158 | 0.156 | 0.158 | 0.185 | 0.175 | 0.165 | 0.167 | 0.181 |  |  |  |  |  |
| **12** | *O. magnapustulosa* | 0.149 | 0.154 | 0.158 | 0.154 | 0.158 | 0.187 | 0.177 | 0.166 | 0.185 | 0.188 | 0.164 |  |  |  |  |
| **13** | *O. myanhesei* | 0.147 | 0.152 | 0.155 | 0.152 | 0.155 | 0.179 | 0.171 | 0.164 | 0.190 | 0.197 | 0.173 | 0.061 |  |  |  |
| **14** | *O. martensii* | 0.147 | 0.153 | 0.158 | 0.153 | 0.158 | 0.185 | 0.173 | 0.162 | 0.185 | 0.188 | 0.167 | 0.044 | 0.060 |  |  |
| **15** | *O. swanbornorum* | 0.150 | 0.145 | 0.147 | 0.145 | 0.147 | 0.177 | 0.174 | 0.161 | 0.181 | 0.174 | 0.174 | 0.056 | 0.064 | 0.065 |  |
