# Supplementary material for: Integrative taxonomic analyses reveal first country records of Occidozygashiwandashanensis Chen, Peng, Liu, Huang, Liao & Mo, 2022 and Hylaranalatouchii (Boulenger, 1899) (Anura, Dicroglossidae, Ranidae) from Vietnam
Source: Biodivers Data J. 2023 Oct 13;11:e109726. doi: 10.3897/BDJ.11.e109726 (PMC10589760; doi:10.3897/BDJ.11.e109726)
Supplement: Supplementary material 3 — Uncorrected (“p”) distance matrix of seven species of Hylarana [file bdj-11-e109726-s003.docx]

Table S3: Uncorrected (“p”) distance matrix showing percentage pairwise genetic divergence 16S between the two sequences of collected samples of *Hylarana* and available sequences of seven species of *Hylarana* in GenBank.

|  | **Species** | **1** | **2** | **3** | **4** | **5** | **6** | **7** | **8** | **9** | **10** | **11** | **12** |
| --- | --- | --- | --- | --- | --- | --- | --- | --- | --- | --- | --- | --- | --- |
| **1** | *H. latouchii* IEBR A.5205 |  |  |  |  |  |  |  |  |  |  |  |  |
| **2** | *H. latouchii* IEBR A.5204 | 0.36 |  |  |  |  |  |  |  |  |  |  |  |
| **3** | *H. latouchii* MT702387 | 2.19 | 2.56 |  |  |  |  |  |  |  |  |  |  |
| **4** | *H. latouchii* MN241431 | 2.56 | 2.56 | 0.90 |  |  |  |  |  |  |  |  |  |
| **5** | *H. latouchii* AB058880 | 2.08 | 2.08 | 2.27 | 2.27 |  |  |  |  |  |  |  |  |
| **6** | *H. latouchii* LC640538 | 2.01 | 2.01 | 1.81 | 2.01 | 0.00 |  |  |  |  |  |  |  |
| **7** | *H. spinulosa* | 2.98–3.43 | 3.17–3.62 | 3.77–4.24 | 3.58–4.05 | 3.84–4.32 | 3.67–3.88 | 0–1.13 |  |  |  |  |  |
| **8** | *H. maosonensis* | 2.4–3.97 | 2.4–4.17 | 3.18–4.21 | 3.13–4.61 | 2.27–4.27 | 2.21–4.12 | 2.53–4.25 | 0–4.44 |  |  |  |  |
| **9** | *H. cubitalis* | 4.76 | 4.76 | 6.46 | 6.24 | 5.32 | 5.33 | 5.61–6.25 | 5.18–6.32 | 0.00 |  |  |  |
| **10** | *H. lacrima* | 8.55–8.67 | 8.96–9.08 | 9.2–9.32 | 9.17–9.29 | 8.65–8.9 | 8.06–8.32 | 9.78–10.79 | 8.89–10.01 | 9.59–10.12 | 0–1.1 |  |  |
| **11** | *H. annamitica* | 7.63–8.91 | 7.83–9.31 | 8.29–9.16 | 8.29–9.16 | 7.19–9.29 | 6.72–8.52 | 8.24–10.1 | 7.02–9.95 | 8.93–10.08 | 9.6–10.24 | 0–3.55 |  |
| **12** | *H. nigrovittata* | 6.15–6.92 | 5.76–7.26 | 6.16–7.11 | 5.79–6.69 | 6.74–7.54 | 6.51–7.36 | 6.59–8.51 | 5.76–7.93 | 7.59–8.38 | 9.95–10.81 | 5.53–6.86 | 0–2.8 |
