# Supplementary material for: Integrative taxonomic analyses reveal first country records of Occidozygashiwandashanensis Chen, Peng, Liu, Huang, Liao & Mo, 2022 and Hylaranalatouchii (Boulenger, 1899) (Anura, Dicroglossidae, Ranidae) from Vietnam
Source: Biodivers Data J. 2023 Oct 13;11:e109726. doi: 10.3897/BDJ.11.e109726 (PMC10589760; doi:10.3897/BDJ.11.e109726)
Supplement: Supplementary material 4 — Measurements of Occidozygashiwandashanensis [file bdj-11-e109726-s004.docx]

Table S4. Measurement (in mm) and proportions of the *Occidozyga shiwandashanensis*.

| *Occidozyga shiwandashanensis* | | | | | | | | | | | |
| --- | --- | --- | --- | --- | --- | --- | --- | --- | --- | --- | --- |
| Vietnam specimens | | | | | | | | China specimens | | | |
| Field no | IEBR A.5200 | IEBR  A.5199 | IEBR A.5201 | IEBR A.5202 | IEBR A.5203 | Min-Max | TB±SD | Min-Max | TB±SD | Min-Max | TB±SD |
| Sex | M | F | F | F | F |  |  | M (n=22) | M (n=22) | F (n=10) | F (n=10) |
| SVL | 28.1 | 38.6 | 36.2 | 37.4 | 39.5 | 36.20-39.5 | 37.93±1.44 | 25.2-33.8 | 29.8±2.3 | 34.9-38.9 | 36.8±1.5 |
| HW | 10.9 | 16.4 | 13.5 | 15.7 | 16.6 | 13.50-16.60 | 15.55±1.42 | 8.8-12.8 | 11.2±1.2 | 12.2-15.7 | 13.0±1.1 |
| HL | 9.4 | 14.6 | 12.1 | 13.5 | 15.1 | 12.10-15.10 | 13.83±1.33 | 7.8-11.8 | 9.6±1.3 | 9.8-14.5 | 11.4±1.4 |
| MN | 7.7 | 11.6 | 10.8 | 11.6 | 13.2 | 10.80-13.20 | 11.80±1.01 | - | - | - | - |
| MFE | 7 | 9.7 | 9.3 | 10.1 | 11.6 | 9.30-11.60 | 10.18±1.00 | - | - | - | - |
| MBE | 4.7 | 5.8 | 5.4 | 6.2 | 7.3 | 5.40-7.30 | 6.18±0.82 | - | - | - | - |
| RL | 3.6 | 4.4 | 3.9 | 4.3 | 4.9 | 3.90-4.90 | 4.38±0.41 | 3.3-4.5 | 3.8±0.3 | 3.6-5.1 | 4.4±0.4 |
| ED | 3.8 | 4.6 | 4.3 | 4.7 | 5.1 | 4.30-5.10 | 4.68±0.33 | 2.4-4.3 | 3.6±0.4 | 3.4-4.6 | 4.0±0.3 |
| NS | 1.7 | 1.8 | 1.4 | 1.9 | 2.1 | 1.40-2.10 | 1.80±0.29 | 1.3-1.9 | 1.5±0.2 | 1.5-2.7 | 2.0±0.3 |
| EN | 1.9 | 2.6 | 2.5 | 2.4 | 2.8 | 2.40-2.80 | 2.58±0.17 | 1.3-2.0 | 1.5±0.2 | 1.5-2.9 | 2.1±0.4 |
| UEW | 2.5 | 2.6 | 2.3 | 2.5 | 2.8 | 2.30-2.80 | 2.55±0.21 | 2.4±0.2 | 2.55±0.21 | 2.0–3.1 | 2.7±0.3 |
| IOD | 1.8 | 2.3 | 2 | 2.2 | 2.4 | 2.00-2.40 | 2.23±0.17 | 1.5±0.3 | 2.23±0.17 | 1.6–2.2 | 1.9±0.2 |
| IND | 2.7 | 3.4 | 2.9 | 3.5 | 3.6 | 2.90-3.60 | 3.35±0.31 | 2.1±0.5 | 3.35±0.31 | 1.5-2.9 | 2.4±0.5 |
| DAE | 3.8 | 4.7 | 4.4 | 4.6 | 4.9 | 4.40-4.90 | 4.65±0.21 | - | - | - | - |
| DPE | 6.1 | 7.5 | 7.2 | 7.6 | 7.8 | 7.20-7.80 | 7.53±0.25 | - | - | - | - |
| FLL | 4.8 | 5.8 | 5.7 | 6.1 | 6.5 | 5.70-6.50 | 6.03±0.36 | - | - | - | - |
| HAL | 11.1 | 14.9 | 13.6 | 13.7 | 14.8 | 13.60-14.90 | 14.25±0.70 | 10.3-13.3 | 11.6±0.8 | 11.9-14.7 | 13.2±0.9 |
| FL1 | 4.6 | 6.3 | 5.9 | 6.2 | 6.4 | 5.90-6.40 | 6.20±0.22 | - | - | - | - |
| FL2 | 3.7 | 5.6 | 5.2 | 5.8 | 5.9 | 5.20-5.90 | 5.63±0.31 | - | - | - | - |
| FL3 | 6.3 | 7.8 | 7.3 | 7.7 | 8.2 | 7.30-8.20 | 7.75±0.37 | - | - | - | - |
| FL4 | 5.1 | 6.6 | 6.2 | 6.4 | 7.1 | 6.20-7.10 | 6.58±0.39 | - | - | - | - |
| FeL | 12.1 | 17.3 | 15.5 | 16.4 | 16.8 | 15.50-17.30 | 16.50±0.76 | 11.4-15.9 | 14.3±1.2 | 15.3-19.9 | 16.7±1.3 |
| TbL | 11.4 | 16.6 | 15.1 | 15.7 | 16.1 | 15.10-16.60 | 15.88±0.63 | 11.5-15.6 | 13.6±1.0 | 15.3-17.9 | 16.2±0.8 |
| TbW | 4.6 | 6.4 | 6.2 | 6.3 | 6.7 | 6.20-6.70 | 6.40±0.22 | - | - | - | - |
| FoL | 17.9 | 21.5 | 21.1 | 21.7 | 23.4 | 21.10-23.40 | 21.93±1.01 | 12.4-15.1 | 13.7±0.7 | 14.3-17.6 | 16.0±0.9 |
| TL1 | 5.1 | 7.1 | 6.8 | 7 | 7.2 | 6.80-7.20 | 7.03±0.17 | 4.2-6.1 | 5.2±0.5 | 5.6-6.8 | 6.0±0.3 |
| TL2 | 7.2 | 10.1 | 9.8 | 10.3 | 10.9 | 9.80-10.90 | 10.28±0.46 | - | - | - | - |
| TL3 | 10.6 | 13.2 | 12.7 | 13.1 | 13.8 | 12.70-13.80 | 13.20±0.45 | - | - | - | - |
| TL4 | 12.3 | 15.2 | 14.6 | 14.9 | 15.7 | 14.60-15.70 | 15.10±0.47 | - | - | - | - |
| TL5 | 9.8 | 12.8 | 12.1 | 12.5 | 13.5 | 12.10-13.50 | 12.73±0.59 | - | - | - | - |
| IMT | 1.5 | 2.0 | 1.7 | 2.2 | 1.7 | 1.7-2.5 | 2.1±0.34 | 1.6-2.7 | 2.1±0.2 | 2.3-2.8 | 2.6±0.2 |
| HL/SVL | 0.33 | 0.38 | 0.33 | 0.36 | 0.38 | 0.33-0.38 | 0.36±0.02 | - | - | - | - |
| HW/SVL | 0.39 | 0.42 | 0.37 | 0.42 | 0.42 | 0.37-0.42 | 0.41±0.02 | - | - | - | - |
| HL/HW | 0.86 | 0.89 | 0.90 | 0.86 | 0.91 | 0.86-0.91 | 0.89±0.02 | - | - | - | - |
| ED/RL | 1.06 | 1.05 | 1.10 | 1.09 | 1.04 | 1.04-1.10 | 1.07±0.03 | - | - | - | - |
| RL/HL | 0.38 | 0.30 | 0.32 | 0.32 | 0.32 | 0.30-0.32 | 0.32±0.01 | - | - | - | - |
| NS/EN | 0.89 | 0.69 | 0.56 | 0.79 | 0.75 | 0.56-0.79 | 0.70±0.10 | - | - | - | - |
| IOD/UEW | 0.72 | 0.88 | 0.87 | 0.88 | 0.86 | 0.86-0.88 | 0.87±0.01 | - | - | - | - |
| FeL/SVL | 0.43 | 0.45 | 0.43 | 0.44 | 0.43 | 0.43-0.45 | 0.44±0.01 | - | - | - | - |
| FLL/SVL | 0.17 | 0.15 | 0.16 | 0.16 | 0.16 | 0.15-0.16 | 0.16±0.01 | - | - | - | - |
| HAL/SVL | 0.40 | 0.39 | 0.38 | 0.37 | 0.37 | 0.37-0.39 | 0.38±0.01 | - | - | - | - |
| FoL/SVL | 0.64 | 0.56 | 0.58 | 0.58 | 0.59 | 0.56-0.59 | 0.58±0.02 | - | - | - | - |
| TbL/SVL | 0.41 | 0.43 | 0.42 | 0.42 | 0.41 | 0.41-0.43 | 0.42±0.01 | - | - | - | - |
| TbL/TbW | 2.48 | 2.59 | 2.44 | 2.49 | 2.40 | 2.40-2.59 | 2.48±0.08 | - | - | - | - |
