# Supplementary material for: Integrative taxonomic analyses reveal first country records of Occidozygashiwandashanensis Chen, Peng, Liu, Huang, Liao & Mo, 2022 and Hylaranalatouchii (Boulenger, 1899) (Anura, Dicroglossidae, Ranidae) from Vietnam
Source: Biodivers Data J. 2023 Oct 13;11:e109726. doi: 10.3897/BDJ.11.e109726 (PMC10589760; doi:10.3897/BDJ.11.e109726)
Supplement: Supplementary material 5 — Measurements of Hylaranalatouchii [file bdj-11-e109726-s005.docx]

Table S3. Measurement (in mm) and proportions of the *Hylarana latouchii*.

|  | *Hylarana latouchii* | | | | | | | | |
| --- | --- | --- | --- | --- | --- | --- | --- | --- | --- |
| Field no | IEBR A.2013.30 | IEBR A.2013.29 | IEBR A. 2013.27 | IEBR A.5205 | IEBR A.5206 | IEBR A.5204 |  |  | IEBR A.2013.41 |
| Sex | M | M | M | M | M | M | Min-Max | TB±SD | F |
| SVL | 51.70 | 48.60 | 49.60 | 49.80 | 51.50 | 49.70 | 48.60-51.70 | 50.15±1.20 | 58.40 |
| HW | 18.20 | 17.40 | 17.60 | 18.20 | 17.80 | 17.90 | 17.40-18.20 | 17.85±0.32 | 19.50 |
| HL | 20.10 | 19.40 | 19.70 | 20.60 | 19.90 | 19.80 | 19.40-20.60 | 19.92±0.41 | 22.80 |
| MN | 17.30 | 16.60 | 16.90 | 17.70 | 17.30 | 17.10 | 16.60-17.70 | 17.15±0.38 | 19.60 |
| MFE | 14.20 | 12.90 | 13.80 | 14.50 | 14.40 | 13.70 | 12.90-14.50 | 13.92±0.59 | 16.20 |
| MBE | 8.70 | 7.80 | 7.50 | 8.90 | 9.10 | 8.30 | 7.50-9.10 | 8.38±0.63 | 9.90 |
| RL | 7.90 | 7.70 | 7.20 | 8.40 | 7.60 | 7.50 | 7.20-8.40 | 7.72±0.41 | 9.20 |
| ED | 7.20 | 6.90 | 6.60 | 7.10 | 6.50 | 6.80 | 6.50-7.20 | 6.85±0.27 | 7.60 |
| NS | 3.40 | 3.30 | 3.40 | 4.00 | 3.20 | 3.30 | 3.20-4.00 | 3.43±0.29 | 4.10 |
| EN | 4.50 | 4.40 | 3.80 | 4.40 | 4.30 | 4.20 | 3.80-4.50 | 4.27±0.25 | 5.10 |
| TD | 4.30 | 4.60 | 4.50 | 4.30 | 4.10 | 4.40 | 4.10-4.60 | 4.37±0.18 | 4.90 |
| TYE | 2.30 | 1.80 | 1.70 | 1.90 | 2.10 | 2.20 | 1.70-2.30 | 2.00±0.24 | 2.40 |
| UEW | 3.90 | 3.90 | 3.80 | 4.20 | 3.80 | 4.10 | 3.80-4.20 | 3.95±0.16 | 4.80 |
| IOD | 4.80 | 4.60 | 4.70 | 4.60 | 4.90 | 5.10 | 4.60-5.10 | 4.78±0.19 | 5.30 |
| IND | 5.50 | 5.40 | 5.40 | 5.60 | 5.20 | 5.60 | 5.20-5.60 | 5.45±0.15 | 6.70 |
| DAE | 8.60 | 8.90 | 8.20 | 9.30 | 8.20 | 9.20 | 8.20-9.30 | 8.73±0.48 | 10.20 |
| DPE | 13.10 | 12.20 | 12.60 | 12.90 | 12.40 | 12.30 | 12.20-13.10 | 12.58±0.35 | 14.60 |
| FLL | 11.30 | 10.40 | 11.20 | 10.80 | 11.50 | 10.70 | 10.40-11.50 | 10.98±0.42 | 12.70 |
| HAL | 24.50 | 23.60 | 23.90 | 24.20 | 22.70 | 23.40 | 22.70-24.50 | 23.72±0.64 | 27.70 |
| FL1 | 10.10 | 9.70 | 9.60 | 9.50 | 9.30 | 10.40 | 9.30-10.40 | 9.77±0.41 | 11.30 |
| FL2 | 9.50 | 8.70 | 9.20 | 9.30 | 8.50 | 9.10 | 8.50-9.50 | 9.05±0.38 | 10.50 |
| FL3 | 12.90 | 12.50 | 12.40 | 13.20 | 12.60 | 12.30 | 12.30-13.20 | 12.65±0.34 | 15.20 |
| FL4 | 11.00 | 10.60 | 10.80 | 10.40 | 10.30 | 10.90 | 10.30-11.00 | 10.67±0.28 | 13.10 |
| NPL | 6.60 | 6.20 | 6.10 | 6.30 | 5.50 | 6.50 | 5.50-6.60 | 6.20±0.39 | - |
| FeL | 24.60 | 22.70 | 23.20 | 24.30 | 24.10 | 23.40 | 22.70-24.60 | 23.72±0.73 | 27.20 |
| TbL | 27.80 | 25.80 | 26.70 | 27.30 | 26.20 | 27.10 | 25.80-27.80 | 26.82±0.74 | 29.70 |
| TbW | 6.20 | 5.30 | 5.60 | 5.40 | 5.20 | 6.10 | 5.20-6.20 | 5.63±0.42 | 5.90 |
| FoL | 37.30 | 35.30 | 35.70 | 36.90 | 34.10 | 35.50 | 34.10-37.30 | 35.80±1.16 | 40.10 |
| TL1 | 8.50 | 7.60 | 7.50 | 8.20 | 7.70 | 7.50 | 7.50-8.50 | 7.83±0.42 | 9.10 |
| TL2 | 12.70 | 10.40 | 11.90 | 12.10 | 12.50 | 11.90 | 10.40-12.70 | 11.92±0.81 | 12.90 |
| TL3 | 18.60 | 16.70 | 18.70 | 18.60 | 18.20 | 18.20 | 16.70-18.70 | 18.17±0.75 | 19.60 |
| TL4 | 26.20 | 23.30 | 23.70 | 25.50 | 25.40 | 25.20 | 23.30-26.20 | 24.88±1.13 | 26.80 |
| TL5 | 18.90 | 17.50 | 17.80 | 18.80 | 18.70 | 18.50 | 17.50-18.90 | 18.37±0.58 | 21.80 |
| HL/SVL | 0.39 | 0.40 | 0.40 | 0.41 | 0.39 | 0.40 | 0.39-0.41 | 0.40±0.01 | 0.39 |
| HW/SVL | 0.35 | 0.36 | 0.35 | 0.37 | 0.35 | 0.36 | 0.35-0.37 | 0.36±0.01 | 0.33 |
| HL/HW | 1.10 | 1.11 | 1.12 | 1.13 | 1.12 | 1.11 | 1.10-1.13 | 1.12±0.01 | 1.17 |
| TD/ED | 0.60 | 0.67 | 0.68 | 0.61 | 0.63 | 0.65 | 0.60-0.68 | 0.64±0.03 | 0.64 |
| ED/RL | 0.91 | 0.90 | 0.92 | 0.85 | 0.86 | 0.91 | 0.85-0.92 | 0.89±0.03 | 0.83 |
| RL/HL | 0.39 | 0.40 | 0.37 | 0.41 | 0.38 | 0.38 | 0.37-0.41 | 0.39±0.01 | 0.40 |
| NS/EN | 0.76 | 0.75 | 0.89 | 0.91 | 0.74 | 0.79 | 0.74-0.91 | 081±0.08 | 0.80 |
| IOD/UEW | 1.23 | 1.18 | 1.24 | 1.10 | 1.29 | 1.24 | 1.10-1.29 | 1.21±0.07 | 1.10 |
| FeL/SVL | 0.48 | 0.47 | 0.47 | 0.49 | 0.47 | 0.47 | 0.47-0.49 | 0.47±0.01 | 0.47 |
| FLL/SVL | 0.22 | 0.21 | 0.23 | 0.22 | 0.22 | 0.22 | 0.21-0.23 | 0.22±0.00 | 0.22 |
| HAL/SVL | 0.47 | 0.49 | 0.48 | 0.49 | 0.44 | 0.47 | 0.44-0.49 | 0.47±0.02 | 0.47 |
| FoL/SVL | 0.72 | 0.73 | 0.72 | 0.74 | 0.66 | 0.71 | 0.66-0.74 | 0.71±0.03 | 0.69 |
| TbL/SVL | 0.54 | 0.53 | 0.54 | 0.55 | 0.51 | 0.55 | 0.51-0.55 | 0.53±0.01 | 0.51 |
| TbL/TbW | 4.48 | 4.87 | 4.77 | 5.06 | 5.04 | 4.44 | 4.44-5.06 | 4.78±0.27 | 5.03 |
